# Nerve MR in the Differential Diagnosis of Neuropathies: A Case Series from a Single Center

**DOI:** 10.3390/jcm12155009

**Published:** 2023-07-30

**Authors:** Carolina Giordano, Maria Ausilia Sciarrone, Francesca Vitali, Angela Romano, Giulia Guerri, Valentina Perlangeli, Simona Gaudino, Marco Luigetti

**Affiliations:** 1 Dipartimento di Diagnostica per Immagini, Radioterapia Oncologica ed Ematologia, Fondazione Policlinico “A. Gemelli” IRCCS, 00168 Rome, Italy; carolina.giordano@guest.policlinicogemelli.it (C.G.); simona.gaudino@policlinicogemelli.it (S.G.); 2 Università Cattolica del Sacro Cuore, 00168 Rome, Italy; a.sciarrone97@gmail.com (M.A.S.); vitali.francesca95@gmail.com (F.V.); giulia.guerri01@icatt.it (G.G.); valentina.perlangeli01@icatt.it (V.P.); 3 Dipartimento Neuroscienze, Organi di Senso e Torace, Fondazione Policlinico “A. Gemelli” IRCCS, 00168 Rome, Italy; angela.romano12@gmail.com

**Keywords:** magnetic resonance neurography, peripheral nervous system, peripheral neuropathy

## Abstract

In the present study, through a case series, we highlighted the role of magnetic resonance (MR) in the identification and diagnosis of peripheral neuropathies. MR neurography allows the evaluation of the course of nerves through 2D and 3D STIR sequences with an isotropic voxel, whereas the relationship between nerves, vessels, osteo-ligamentous and muscular structures can be appraised with T1 sequences. Currently, DTI and tractography are mainly used for experimental purposes. MR neurography can be useful in detecting subtle nerve alterations, even before the onset of symptoms. However, despite being sensitive, MR neurography is not specific in detecting nerve injury and requires careful interpretation. For this reason, MR information should always be supported by instrumental clinical tests.

## 1. Introduction

Peripheral neuropathy is a general term that indicates any disorder of the peripheral nervous system.

Its prevalence in the general population ranges from 1% to 7%, with higher rates among those older than 50 years [1].

The peripheral nerves are susceptible to a variety of toxic, inflammatory, hereditary, infectious, and parainfectious factors that can impair their health and function. Diabetic peripheral neuropathy is the most common form of neuropathy in Europe and worldwide. The prevalence of peripheral neuropathy is estimated to be between 6% and 51% among adults with diabetes depending on age, duration of diabetes, glucose control, and type 1 versus type 2 diabetes [2].

Peripheral neuropathies may be the result of primarily demyelinating damage or primarily axonal injury and can present acutely or with progressive and chronic onset [3]. According to the anatomic region and distribution across the peripheral nervous system, they are classified as mononeuropathy, mononeuropathy multiplex and polyneuropathy [1].

The term mononeuropathy implies a focal lesion of a single peripheral nerve. The usual causes are trauma, focal compression, and entrapment. The most common mononeuropathy is carpal tunnel syndrome caused by entrapment of the median nerve in the carpal tunnel. Mononeuropathy multiplex describes the involvement of multiple separate noncontiguous peripheral nerves either simultaneously or serially. The pattern of nerve involvement is random, multifocal, and frequently evolves quickly. Often, mononeuropathy multiplex is part of the clinical picture of vasculitis, systemic disorders associated with diseases such as polyarteritis nodosa, Churg–Strauss disease, or one of the connective tissue disorders, but it can be vasculitis confined to peripheral nerves. Other systemic diseases such as sarcoidosis, lymphoma, carcinoma, leprosy, Lyme disease, HIV infection, and cryoglobulinemia can also present as a mononeuropathy multiplex [4]. Polyneuropathy is characterized by the simultaneous malfunction of many peripheral nerves. The most common variety of polyneuropathy is distal symmetrical polyneuropathy. In this setting, nerve fibers are believed to be affected in a length–dependent way. The prototype is chronic sensory and motor polyneuropathy associated with diabetes mellitus [3]. Based on an etiopathogenetic approach, peripheral neuropathies can be divided into genetic and acquired forms. The most significant part of genetic polyneuropathies includes variants of Charcot–Marie–Tooth disease [5]. About 70–80% of these patients have a 1.5 Mb duplication of the PMP22 gene, localized on chromosome 17p11.2, thus leading to three copies of the gene. It codes for peripheral myelin protein 22 (PMP22); the duplication leads to an over-expression of PMP22, influencing myelination and affecting motor and sensory functions [6].

Another genetic disorder associated with neuropathy is hereditary transthyretin amyloidosis (ATTRv, v for variant). ATTRv is the most common form of hereditary amyloidosis that can present as a progressive, axonal sensory autonomic and motor neuropathy or as an infiltrative cardiomyopathy [7].

Peripheral neuropathy is also a common manifestation in mitochondrial diseases, varying from a subclinical finding in a multisystem syndrome to a severe, even isolated manifestation in some patients [8].

Acquired neuropathies include immune-mediated diseases such as Guillan–Barrè syndrome (GBS) and chronic inflammatory demyelinating polyradiculoneuropathy (CIDP). Their typical clinical features are proximal and distal limb weakness, sensory loss and generalized areflexia.

GBS and CIDP share many symptoms and signs in the acute phase of the disease. It has been reported that 16% of patients with CIDP have rapidly progressive weakness, with a nadir within 8 weeks from the onset of the disease, which is followed by a chronic course [9]. 

These patients are considered to have acute-onset CIDP (A-CIDP). Diagnosing peripheral neuropathy requires careful clinical evaluation, laboratory tests, and electrodiagnostic studies or nerve biopsy if the diagnosis remains unclear. 

Lumbar puncture and CSF analysis may help diagnose Guillain–Barré syndrome and chronic inflammatory demyelinating neuropathy. Electrodiagnostic studies, including nerve conduction studies and electromyography (EMG), can differentiate whether symptoms are caused by primary axonal or demyelinating damage; this distinction is often relevant to treatment choices and prognosis [10]. 

The electrodiagnostic studies also provide information about the different types of demyelination (uniform vs. multifocal) and the pattern of nerve involvement. 

Imaging in peripheral nerve pathologies, in most cases, integrates clinical history/examination, EMG, and nerve conduction studies (NCS) with spatial and morphological information of the pathology; the role of imaging is even more relevant in cases of electrodiagnostic studies that are indeterminate or not feasible due to inaccessible nerves or dermatological conditions. The most used methods for nerve imaging are ultrasound (US) and MR [11]. Between the two methods, ultrasound examination of the nerve certainly has the advantage of being a faster and cheaper imaging method and makes some essential dynamic tests possible, for example, in cases of nerve entrapment. 

However, for studying deeper and proximal portions of the nerves—such as the deep area of the sciatic nerve—and cranial nerves, which cannot be explored through US, the use of MR is suggested, which is also the reference examination for the study of brachial and lumbosacral plexuses. MR also has the advantage of simultaneous exploration of adjacent nerves and muscles; it can detect muscle denervation, which leads to muscle cell edema. 

In the last 15 years, thanks to technological developments, MR neurography has provided increasingly important information in investigating peripheral neuropathies [12,13]. In addition, 3D MR can assess the disease location and may evaluate the size, symmetry, signal intensity, and enhancement variations along plexuses and nerve trunks’ course.

Regardless of the underlying etiology, MR changes in pathological nerves result from an increased water content in the epineurial space following damage to the blood–nerve barrier, blockage of axoplasmic flow, inflammation, and distal Wallerian degeneration. These alterations manifest themselves on MR as hyperintensity in T2w and thickening of the nerve itself only if the examination is conducted with a rigorous technique and high-field magnets (1.5 T/3 T) [14]. 

Diffusion tensor imaging (DTI) is a quantitative MR technique that measures both the direction and magnitude of proton diffusion. Thanks to its parameters, it can detect microstructural nerve anomalies; there is some evidence that fractional anisotropy (a quantitative DTI parameter) is related to axonal damage and clinical impairment, as pathological conditions such as axoplasmic flow blockage or venous congestion and distal Wallerian degeneration lead to potential space widening between the axons and the surrounding cover, resulting in increased proton scattering and decreased fractional anisotropy values) [14].

Using MR, documenting the signs of denervation in the muscles innervated by the nerve being evaluated is also possible. 

Peripheral nerve imaging is widely used in oncology and trauma, while its use in peripheral neuropathies is minimal [15].

This paper will give an overview of the current MR techniques for studying peripheral nerves, in addition to showing some clinical cases in which MR imaging has supported or directed the clinical diagnosis of peripheral neuropathies. Through the discussion of clinical cases, we aim to demonstrate the usefulness of MR neurography, if well conducted, in diagnosing peripheral neuropathies, using it as support to traditional diagnostic schemes.

## 2. MR Neurography

The demand for MR studies of the peripheral nervous system is growing in peripheral neuropathies.

The MR study of the peripheral nervous system must recognize the detailed knowledge of the anatomy of the region of the plexuses extended from the spine to the terminal banks and peripheral nerves. 

The acquisition must be rigorous, with dedicated sequences that guarantee full anatomical detail and effective fat signal suppression. 

The anatomical complexity of the plexus region presents a technical challenge in MR due to magnetic susceptibility and pulsatility artifacts of the heart and great vessels in the brachial plexus and bowel movement for the lumbosacral plexus. 

The RM study of the brachial and lumbosacral plexus at 1.5 and 3 T involves using 3D STIR sequences with isotropic voxels, with a single volume oriented on the coronal plane, fundamental for multiplanar (MPR) and maximum intensity projection (MIP) reconstructions that are essential for evaluating the course of the roots, trunks, and cords [16]. 

Neuropathies manifest themselves as diffuse, enlargement and T2-weighted signal hyperintensity of nerves; the difficulty in distinguishing small vessels from nerve structures has recently been overcome with the use of intravenous administration of gadolinium, during 3D STIR sequence acquisition, for vascular signal suppression [17]. 

The coronal T1 sequences provide information on the anatomical relationships between the nervous plexus, vessels, and osteo-ligamentous and muscular structures. 

Two-dimensional axial high-resolution T1-weighted and fat-suppressed T2-weighted images serve as the cornerstone in MR interpretation for the conservative evaluation of imaging characteristics of larger peripheral nerves (such as the sciatic and median nerves). They make it possible to evaluate the signal intensity, course, caliber, fascicular pattern, and size of the nerves [14,18]. 

Normal nerves show intermediate (muscle-like) signals on T1-weighted images and minimally increased signals on T2-weighted images, depending on the amount of endoneurial fluid, the number of collagen fibers contained in the perineurium and endoneurium, and suppression of background fat. The epineurium appears as a thin hypointense border. Furthermore, it is possible to recognize the fascicular pattern of the nerves characterized by a slightly higher signal intensity than the surrounding perineural and epineural tissue due to the presence of endoneurial fluid [14,18]. 

In cases of neuropathy, the nerve signal increases abnormally, approaching the fluid signal of adjacent vessels; on T2-weighted imaging, the fascicular pattern is lost, or hypertrophy of some or all of the fascicles may be observed [19]. 

T1-weighted images after gadolinium are indicated in specific indications, such as suspected neoplasm or inflammatory polyneuropathies, as normal nerves possess the blood–neural barrier, so they have no enhancement [18]. 

DTI allows us to analyze the microstructure and function of nerves and provide information on the trajectory of the fibers. It also makes it possible to calculate quantitative parameters such as the apparent diffusion coefficient (ADC), which describes the average diffusivity and the fractional anisotropy. Various nerve abnormalities and injuries, such as trauma, entrapment, tumor, and inflammation, can lead to decreased fractional anisotropy and increased ADC values [14].

MR also provides information on muscle denervation. In the acute phase of muscle denervation, inflammatory phenomena will lead to an increase in extracellular fluid, which determines an increase in signals on T2w images. In the subacute and chronic phases, a progressive replacement of muscle with fat will be observed, which is detectable on MR as T1 hyperintensity [15]. 

## 3. Clinical Vignette and Image(s)

### 3.1. Clinical Vignette and Image(s)#1: MR Neurography Has Guided the Clinical Diagnosis of Peripheral Neuropathies

Patient history: a 60-year-old patient presented with stocking paresthesias, gait instability, left ptosis, and facial paralysis for three days. 

Clinical and laboratory examination: the neurological examination also revealed dysarthria, ophthalmoparesis, left tongue deviation, and absent deep tendon reflexes. No muscle weakness or autonomic failure occurred during the clinical course. NCS documented mild demyelinating sensory and motor neuropathy. Specifically, the study showed increased mean F-wave latency of the tibial and ulnar nerves bilaterally, and reduced motor conduction velocity in all nerves tested (peroneal, median, and ulnar nerves bilaterally). CSF examination was unremarkable.

First clinical hypothesis: Guillain–Barré syndrome (GBS), for which intravenous immunoglobulin (IVIg) was started with prompt recovery.

Imaging technique: brain and spinal cord MR with gadolinium. MR neurography of the cranial nerves, brachial and lumbosacral plexus. Nerve ultrasound was not used in the diagnostic workup.

**Figure 1:**
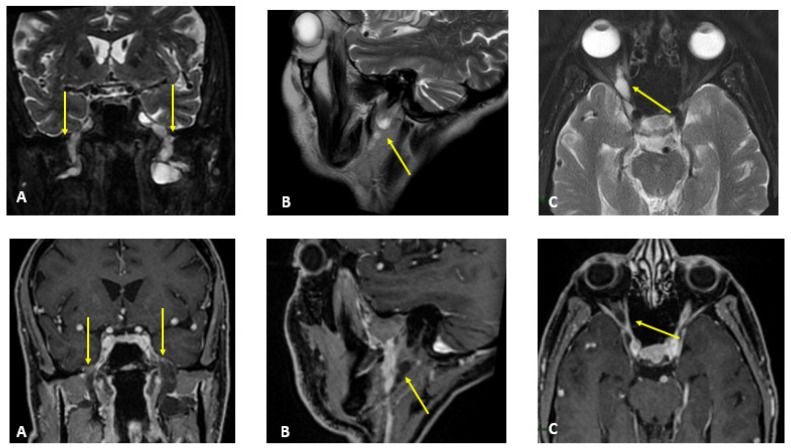
MR T2-weighted (**top**) and T1-weighted fat post-contrast contrast images (**bottom**). The images in (**A**) are in the coronal plane, those in (**B**) are in the sagittal plane and those in (**C**) are in the axial plane. The images show thickened and pathological cranial nerves with a cystic appearance, high signals in T2w images and almost no contrast enhancement. In both (**A**), yellow arrows indicate pathological V3 branches of the trigeminal nerve (transverse diameter after the foramen ovale: left 3.8 mm; right 10 mm); in both (**B**), yellow arrows indicate pathological facial nerve in the intraparotid course (transverse diameter in intraparotid course: left 6.7 mm vs. right 2.2 mm); in both (**C**), yellow arrows indicate pathological right V1 branch of the trigeminal nerve (transverse diameter after superior orbital foramen: right 6.9 mm vs. left 2.3 mm).

**Figure 2:**
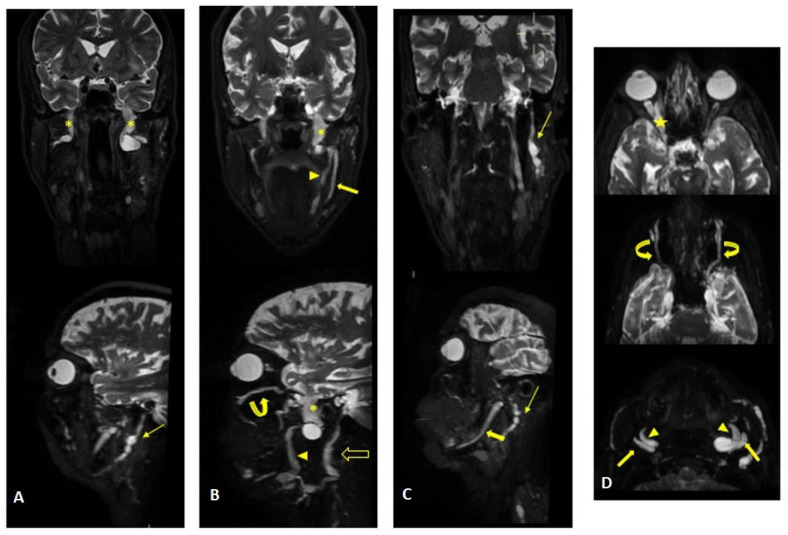
MR (multiplanar reconstructions) of 3D cube neurography images show pathological, thickened cranial nerves along their extracranial course, with some cystic-like components. The images show the involvement of trigeminal branches V1 in ((**D**)—yellow star), V2 in ((**B**,**D**)—curved arrows with a transverse diameter after inferior orbital foramen: left 1.8 mm; right 2.6 mm) and V3 from its passage in the foramen ovale (asterisks in (**A**,**B**)) to its lingual (arrowheads in (**B**,**D**)) and alveolar divisions (thick arrows in (**B**–**D**)); the involvement of the left facial nerve in its intraparotid course (thin arrows in (**A**,**C**)) and the hypoglossal nerve (open arrow in (**B**), transverse diameter at C1 level: left 4.6 mm; right 2.1 mm).

**Figure 3:**
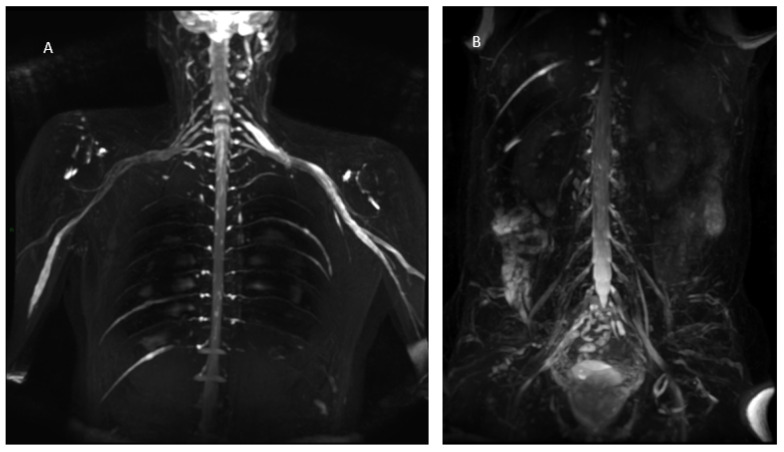
MIP (Maximum Intensity Projection) reconstructions of the 3D cube nerve MR sequence of the brachial (**A**) and lumbosacral (**B**) plexus showing a pathological and asymmetric thickening and increase in T2 signal representation of them.

**Figure 4:**
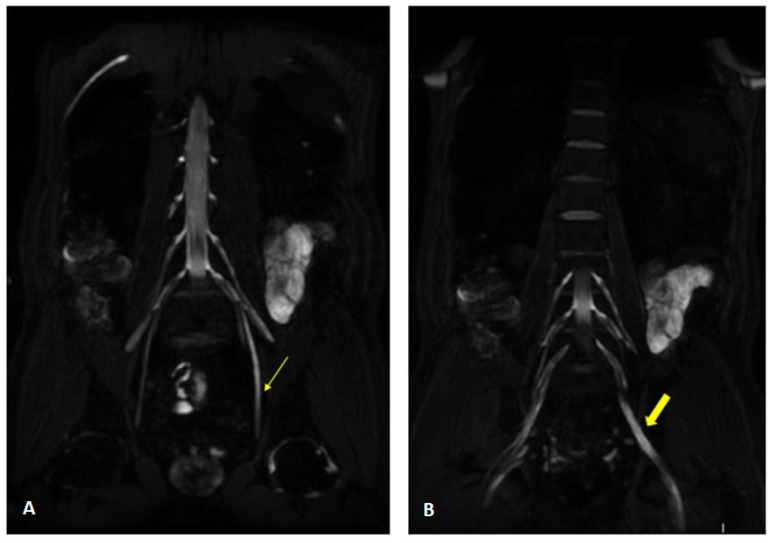
MR 3D cube nerve images show thickening and T2 hyperintensity of the left obturator nerve (thin arrow in **A**) and lumbosacral trunk (thick arrow in **B**) compared to the contralateral one (transverse diameter on left L5 root 7.7 mm (n.v. * 5.99 ± 0.66) and on left S1 root 9.1 mm (n.v. * 5.27 ± 0.53). * Normal values (n.v.) according to the recent study of Su X et al. [20].

**Figure 5:**
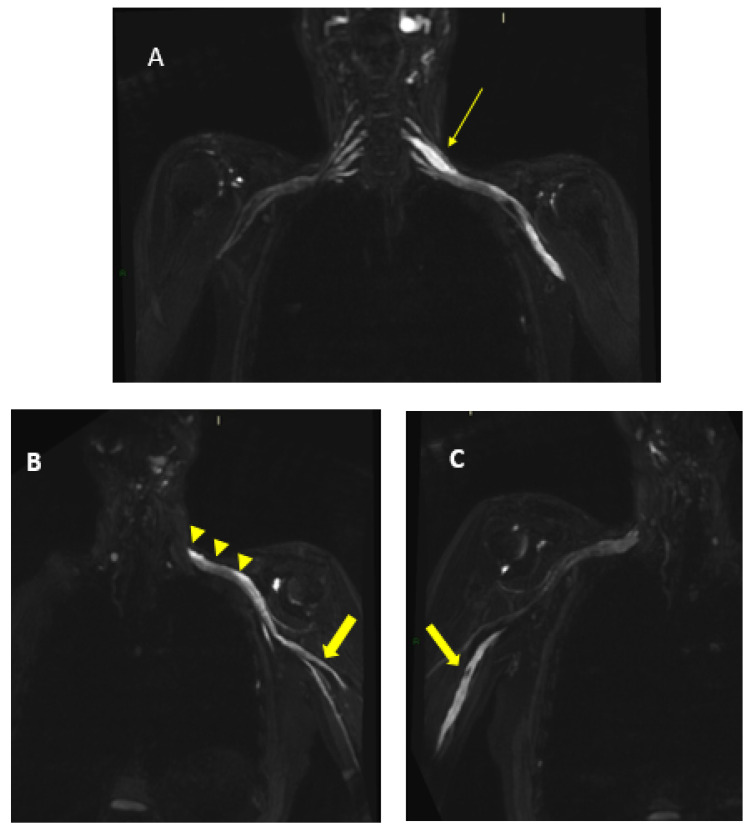
MR 3D cube nerve images show thickening and T2 hyperintensity of brachial plexus and in particular in (**A**), we can observe a pathologic left C6 root (thin arrow transverse diameter of C6 left root 10.3 mm; n.v. * 4.57 ± 0.51); in B, we can observe the involvement of left trunks and cords (arrowheads) and in (**B**,**C**), we can observe the involvement of the peripheral nerves of the bilateral arms (thick arrows). * Normal values (n.v.) according to the recent study of Su X et al. [20].

MR findings: brain and spinal MR were negative for pathological contrast enhancement of the roots of the cauda equina and radicles emerging from the spinal cord. Instead, swellings along the extracranial course of some V and VI cranial nerve branches had been reported (Figure 1). It was decided to integrate the study with MR neurography of the cranial nerves and the lumbosacral and brachial plexuses, which showed diffuse hypertrophy of the cranial nerves, and in particular of the oculomotor, trigeminal, facial, and hypoglossal (Figure 2) nerves with bilateral associations and asymmetric hypertrophy of the lumbosacral (Figure 3 and Figure 4) and brachialis (Figure 3 and Figure 5) plexuses.

Interpretation: a genetic panel for demyelinating Charcot–Marie–Tooth was performed and was reported negative. Based on the clinical course and thanks to the MR findings, the hypothesis of chronic inflammatory demyelinating polyneuropathy (CIDP) with acute onset was considered.

Treatment: IVIg and oral steroid therapy were started. At the six-month follow-up, the patient was completely cured.

### 3.2. Clinical Vignette and Image(s)#2: MR Neurography Supported the Clinical Diagnosis of Peripheral Neuropathies

Patient history: a 70-year-old man began experiencing paresthesias in his lower limbs at the age of 64. The condition slowly progressed over the following two years. Transthoracic echocardiogram showed a mild hypertrophic cardiomyopathy (interventricular septal thickness 13 mm). Clinical history revealed previous bilateral surgery for carpal tunnel syndrome.

Clinical and laboratory examination: axonal sensory-motor polyneuropathy, with a Neuropathy Impairment Score (NIS) of 48 and a Norfolk Quality of Life-Diabetic Neuropathy (Norfolk QOL-DN) score of 45. The NCS showed reduced amplitude of sensory and motor potentials in the upper and lower limbs with normal conduction velocity associated with bilateral mild carpal tunnel syndrome.

First clinical hypothesis: transthyretin familial amyloid polyneuropathy. Sequence analysis of TTR gene confirmed the presence of the F64L (p. Phe84Leu) variant.

Imaging technique: MR neurography of brachial and lumbosacral plexus. MR neurography of sciatic nerves. Nerve ultrasound was not used in the diagnostic workup.

**Figure 6:**
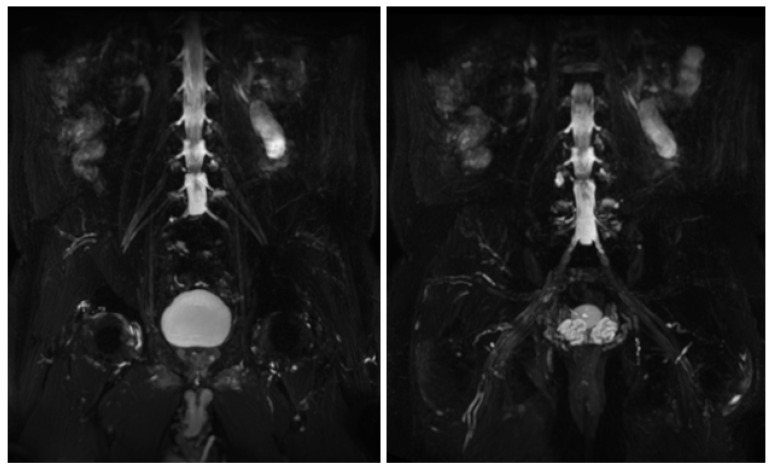
MIP (Maximum Intensity Projection) reconstructions of the 3D cube nerve MR sequence of the lumbosacral plexus show regular size and signal intensity of lumbar and sacral roots.

**Figure 7:**
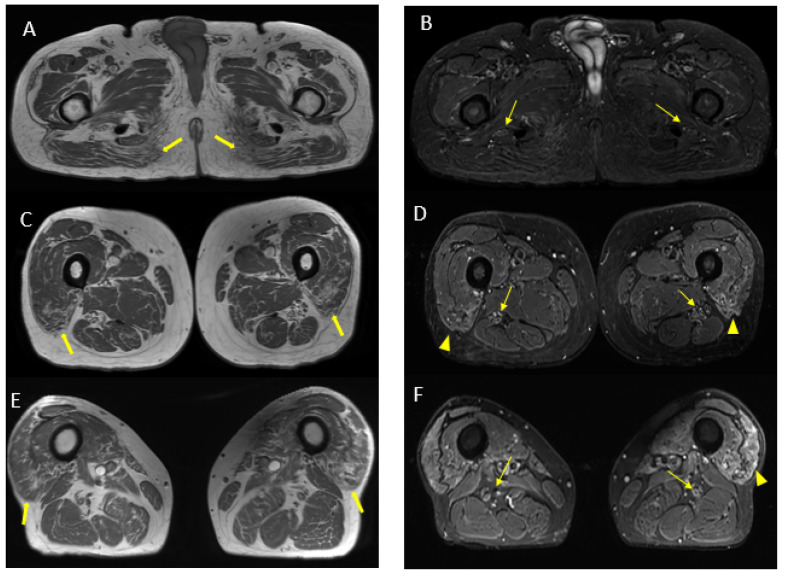
Axial 2D MR of sciatic nerve; on the left are the T1w images and on the right are the T2 STIR images. Bilateral and symmetrical sciatic nerve T2 enlargement and fascicular hyperintensity at the level of the gluteal region (thin arrows (**B**); CSA of sciatic nerve of 103 mm^2^ on the right; 90.1 mm^2^ on the left) and at the mid-thigh (thin arrows in (**D**,**F**)). Mixed, acute and chronic denervation of the muscles of the anterolateral compartment of the thigh with denervation edema (hyperintensity in STIR, arrowheads in (**D**,**F**)) and fat replacement (thick arrows in (**C**,**E**)). Mainly chronic denervation of the gluteal muscles (hyperintensity in T1w, thick arrows in (**A**)).

MR findings: regular size and signal intensity of lumbar and sacral roots (Figure 6). Bilateral and symmetrical sciatic nerve T2 enlargement and fascicular hyperintensity (Figure 7). Mixed acute and chronic denervation of the muscles of the anterolateral compartment of the thigh. Mainly chronic denervation of the gluteal muscles (Figure 7). 

Interpretation: The MR findings are consistent with the amyloid deposits between the fascicles of the sciatic nerve, supporting the clinical diagnosis.

Treatment: gene-silencing therapy was started, resulting in substantial clinical stability after two years of follow-up.

### 3.3. Clinical Vignette and Image(s)#3: MR Neurography Supported the Clinical Diagnosis of Peripheral Neuropathy and Showed Nerve Changes That Precede Overt Clinical Symptoms

Patient history: a 56-year-old man presented with gait disturbance for two years, due to slowly progressing muscle weakness of the left lower limb.

Clinical and laboratory examination: neurological examination revealed left lower limb hyposthenia, evaluated as 3/5 in gastrocnemius muscle and 1/5 in intrinsic foot muscle, absent left Achilles tendon reflex and hypoesthesia of the left foot sole. The patient was unable to walk on his left toes. NCS documented axonal neuropathy of the left sciatic nerve. EMG showed denervation signs (fibrillation potentials and positive sharp waves) in the left lower limb muscles, especially of the gastrocnemius muscle, long head and short head of biceps femoris muscle and semimembranosus muscle.

First clinical hypothesis: left sciatic mononeuropathy.

Imaging technique: MR neurography of brachial and lumbosacral plexus. MR neurography of sciatic and tibial nerves. Nerve ultrasound of the left lower limb.

**Figure 8:**
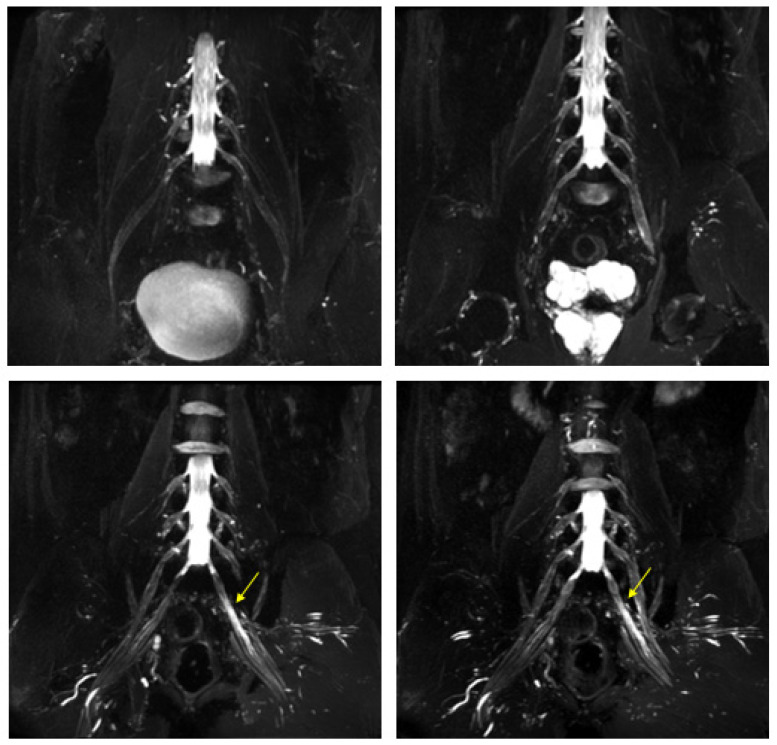
MIP (Maximum Intensity Projection) reconstructions of the 3D cube nerve-MR sequence of the lumbosacral plexus documented a moderate and diffuse increase in thickness and signal intensity on T2W images of the lumbar and sacral roots; the left S1root (arrows) appears to be pathological (thickened and markedly hyperintense in T2w). Transverse diameter S1 roots (n.v. * 5.27 ± 0.53) left 8.4 mm; right 8.2 mm). * Normal values (n.v.) according to the recent study of Su X et al. [20].

**Figure 9:**
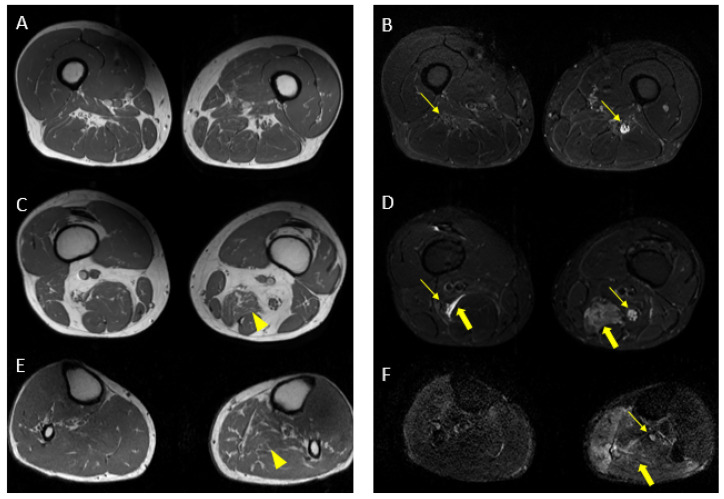
Pathological alterations of the left sciatic nerve (thin arrows in (**B**,**D**)), characterized by a significant increase in volume (CSA in the upper third of the thigh of about 123 mm^2^) with associated fascicular hypertrophy and an increase in signals in T2w images, especially in the height of the middle and lower third of the thigh and with extension to the tibial nerve (thin arrow in (**F**)), up to the lower third of the leg. Also, in the right sciatic nerve, there are some alterations (thin arrows in (**B**,**D**), much more tenuous than the contralateral nerve, characterized by an increase in the thickness of the nerve (CSA at the upper third of the thigh of about 110 mm^2^), a moderate increase signal in T2w images and fascicular hypertrophy, without evident tibial nerve signal alterations. Mixed denervation, acute and chronic, of left and right semimembranosus and posteromedial compartment muscles of the left leg (muscle hyperintensity and fascial edema in STIR, thick arrows in (**D**,**F**)) and fat replacement (muscle bundle atrophy with replacement of hyperintense tissue at T1W in (**A**,**C**,**E**), more evident in some areas: arrowheads in (**C**,**E**)).

MR findings: increase in thickness and signal intensity in T2 W images of lumbar and sacral roots, particularly in the left S1 root that appears to be pathological (Figure 8). Pathological alterations of the left sciatic nerve, characterized by a significant increase in volume with associated fascicular hypertrophy and an increase in signals in the T2w images, especially at the middle and lower thigh and extending in the leg to the tibial nerve. An increase in nerve thickness, a moderate signal increase on T2w images, and fascicular hypertrophy without evident alterations of the tibial nerve signal characterized initial and subtle alterations in the right sciatic nerve. Signs of acute and chronic muscle denervation in the left leg and thigh and initials in the right leg (Figure 9). 

Nerve ultrasound findings: diffuse hypertrophy of the left sciatic nerve (maximum cross-sectional area 110 mm^2^ vs. 80 of the contralateral nerve) and of the tibial nerve at the popliteal level.

Interpretation: MR neurography study confirmed left sciatic and tibial nerve pathology; however, it also showed alterations of the lumbo-sacral plexus and initial involvement of the right sciatic nerve, suggesting a condition of polyradiculoneuropathy. In the light of MR and clinical data, a diagnosis of loco-regional variant CIDP was made. CSF analysis confirmed an increased protein level.

Treatment: successful steroid therapy for two months. The patient experienced clinical worsening after steroid suspension and this therapy was immediately restarted.

## 4. Discussion

The clinical cases presented aim to provide a practical example of the use of MR neurography in diagnosing peripheral neuropathies and the rationale behind its use. 

MR neurography is widely used in traumas to evaluate the extent of the damage and in tumoral lesions to establish dimensions, extension, and relationships with the surrounding structures to support pre-surgical planning. Traditionally, in neuropathies, the diagnostic gold standard is represented by clinical neurological examination supplemented by nerve conduction studies; the MR study was performed to exclude expansive lesions or entrapment phenomena that could underlie the neurogenic suffering [21].

With increasingly sophisticated techniques, MR has been shown to provide images rich in contrast, high resolution, and more quantitative features that could be biomarkers for peripheral nerve pathology in patients with neuropathies [22]. The clinical cases we have presented are a practical demonstration of the usefulness of MR neurography in neuropathies. 

In clinical Vignette #1, the acute onset of peripheral neuropathies, in cases of doubtful lumbar puncture and negative brain and spine MR with gadolinium, would have been oriented towards Guillain–Barré syndrome; however, due to MR neurography, a picture of polyradiculoneuropathy emerged with involvement of the cranial nerves and brachial and lumbar plexus compatible with an inflammatory immunologically mediated form such as CIDP variants, which, even if in a low percentage, can manifest itself with acute onset. 

In clinical Vignette #2, MR neurography showed alterations of the sciatic nerve related to the presence of amyloid deposits between the fascicles with associated signs of muscle denervation in a patient with peripheral neuropathy hereditary transthyretin amyloidosis [23,24,25]. 

The MR alterations of the nerve can be evident even before the onset of frank symptoms, as in Clinical Vignette #3, where mononeuropathy was clinically detected. At the same time, the MR uncovered a condition of asymmetric bilateral polyradiculoneuropathy. Indeed, the criticality of MR is that the increase in T2w signals of the nerve is a sensitive but non-specific marker of nerve injury that requires careful interpretation and consideration of different lesion models; for this reason, the support of instrumental clinical examinations is necessary [26,27]. 

Imaging is in continuous technological progress; new possibilities, such as artificial intelligence, are yet to be explored in nerve imaging, and further studies are needed [28,29,30].

## 5. Conclusions

Historically, the evaluation of peripheral neuropathies relied exclusively on neurophysiology and clinical examination to determine the exact location and nature of the pathology. However, with the progress in the field of neurography imaging, new scenarios have opened up; with MR, it is, in fact, possible to identify pathological nerves with different types of “patterns”, which, in association with clinical and instrumental tests, can be of support and in some cases also decisive in the formulation of the diagnosis.

## Data Availability

Data are available from the corresponding author upon reasonable request.
